# Final overall survival analysis of the phase 3 HERITAGE study demonstrates equivalence of trastuzumab-dkst to trastuzumab in HER2-positive metastatic breast cancer

**DOI:** 10.1007/s10549-021-06197-5

**Published:** 2021-06-14

**Authors:** Hope S. Rugo, Eduardo J. Pennella, Unmesh Gopalakrishnan, Miguel Hernandez-Bronchud, Jay Herson, Hans Friedrich Koch, Subramanian Loganathan, Sarika Deodhar, Ashwani Marwah, Alexey Manikhas, Igor Bondarenko, Guzel Mukhametshina, Gia Nemsadze, Joseph D. Parra, Maria Luisa T. Abesamis-Tiambeng, Kakhaber Baramidze, Charuwan Akewanlop, Ihor Vynnychenko, Virote Sriuranpong, Gopichand Mamillapalli, Sirshendu Roy, Eduardo Patricio Yanez Ruiz, Abhijit Barve, Adolfo Fuentes-Alburo, Cornelius F. Waller

**Affiliations:** 1 University of California San Francisco Helen Diller Family Comprehensive Cancer Center, San Francisco, CA USA; 2 Viatris Inc, Canonsburg, PA USA; 3 Present Address: MaxCyte, Inc, Gaithersburg, MD USA; 4 Viatris Inc, Bangalore, India; 5 GénesisCare Corachan, Barcelona, Spain; 6 Bloomberg School of Public Health, Johns Hopkins University, Baltimore, MD USA; 7 Viatris Inc, Hannover, Germany; 8 Biocon Research Limited, Bangalore, India; 9 City Clinical Oncology Dispensary, Saint Petersburg, Russia; 10 Dnipropetrovsk State Medical Academy, Dnipropetrovsk, Ukraine; 11 Regional Clinical Oncological Center, Kazan, Russia; 12 Institute of Clinical Oncology, Tbilisi, Georgia; 13 St Luke’s Medical Center, Quezon City, Philippines; 14 Cardinal Santos Medical Center, Manila, Philippines; 15 Golden Fleece 21 Century Health House Ltd, Tbilisi, Georgia; 16 Siriraj Hospital, Bangkok, Thailand; 17 Sumy State University, Sumy, Ukraine; 18 King Chulalongkorn Memorial Hospital, Chulalongkorn University, Bangkok, Thailand; 19 City Cancer Center, Vijayawada, India; 20 Curie Manavata Cancer Centre, Nasik, India; 21 Universidad de la Frontera, Temuco, Chile; 22 Department of Haematology, Oncology and Stem Cell Transplantation, University Medical Centre Freiburg and Faculty of Medicine, University of Freiburg, Freiburg, Germany

**Keywords:** Biosimilar, Metastatic breast cancer, Trastuzumab, Overall survival

## Abstract

**Purpose:**

The phase 3 HERITAGE trial demonstrated that the biosimilar trastuzumab-dkst is well tolerated with similar efficacy (measured by overall response rate [ORR] and progression-free survival [PFS]) compared with originator trastuzumab combined with taxane followed by monotherapy in patients with HER2-positive metastatic breast cancer (MBC). Herein, we present final overall survival (OS) from HERITAGE.

**Methods:**

HERITAGE is a multicenter, double-blind, randomized, parallel-group study. Patients were randomized 1:1 to receive trastuzumab-dkst or trastuzumab plus taxane followed by continued monotherapy until disease progression. Overall survival was to be assessed at 36 months or after 240 deaths, whichever occurred first, as observed from time of randomization of last patient.

**Results:**

At the final analysis (36 months), 242 patients in the intention-to-treat population had died during the study: 116 and 124 in the trastuzumab-dkst and trastuzumab groups, respectively, and 1 untreated patient from each treatment group. Median OS by Kaplan–Meier analysis was 35.0 months with trastuzumab-dkst and 30.2 months with trastuzumab. Evaluation of PFS showed a median of 11.1 months in both treatment groups. No new safety concerns were reported from week 48 until the end of the survival follow-up.

**Conclusion:**

This is the first phase 3 trial of a trastuzumab biosimilar to report long-term survival data similar to originator trastuzumab in patients with MBC. The comparable long-term OS between the trastuzumab-dkst and originator trastuzumab groups further supports the similarity of trastuzumab-dkst with originator trastuzumab and establishes trastuzumab-dkst as a safe and effective treatment option for patients with HER2-positive MBC.

ClinicalTrials.gov NCT02472964; 6/16/2015

**Supplementary Information:**

The online version contains supplementary material available at 10.1007/s10549-021-06197-5.

## Introduction

Monoclonal antibodies (mAbs) against tumor-associated markers have been established as safe and effective cancer therapies for several decades [1–4]. Despite therapeutic success, global access to mAbs is limited by the high costs associated with biologic therapies [5–7]. Because of these limitations, there has been an increasing interest in the development of biosimilar agents to provide cost-effective alternatives to expensive biologic cancer therapies [6, 8].

Worldwide, breast cancer is one of the most frequently diagnosed cancers in women, accounting for > 2 million new cancer cases in 2018 [9]. The oncoprotein HER2 is amplified in 15% to 30% of invasive breast cancers (HER2-positive), leading to uncontrolled cell proliferation and tumorigenesis [10]. Trastuzumab (Herceptin®; Genentech Inc, South San Francisco, CA), a humanized IgG1 mAb directed against HER2, was initially approved in 1998 in the United States for the treatment of HER2-overexpressing breast cancer [11]. Combined with chemotherapy, trastuzumab has been associated with significantly improved overall survival (OS) and progression-free survival (PFS), higher overall response rate (ORR), and longer duration of response (DR) in patients with HER2-positive metastatic breast cancer [12].

With the recent patent expirations of trastuzumab in the European Union (2014) and United States (2019), several trastuzumab biosimilars have been developed [13, 14]. Recently, the trastuzumab biosimilar trastuzumab-dkst (Ogivri®; Viatris Inc, Canonsburg, PA) was approved by the US Food and Drug Administration (FDA) and the European Medicines Agency (EMA) for the treatment of HER2-overexpressing breast cancer and metastatic gastric or gastroesophageal junction cancer [13, 15]. Approval was based on robust analytical and pharmacokinetic (PK) data, as well as the results of the HERITAGE trial, a phase 3 study comparing the safety, tolerability, and efficacy of trastuzumab-dkst and trastuzumab in patients with HER2-positive metastatic breast cancer [16–18].

As previously reported, results from the phase 3 HERITAGE trial demonstrated that the ORR was equivalent between trastuzumab-dkst and trastuzumab each in combination with taxane-based chemotherapy at 24 weeks [16]. After combination therapy, patients with stable disease continued their assigned monotherapy until disease progression, unacceptable toxicity, or death, whichever occurred first. No significant differences in ORR, PFS, or interim OS were observed between the trastuzumab-dkst and trastuzumab groups at week 48. Week 24 ORR was highly correlated with PFS at week 48, indicating similarity of the 2 therapies and supporting the use of ORR as a valid endpoint in clinical trials for metastatic breast cancer [19]. We now present the results of the final OS analysis after 36 months and overall safety analysis of the HERITAGE trial.

## Methods

This was a multicenter, double-blind, randomized, parallel-group, phase 3 study (ClinicalTrials.gov, NCT02472964) in patients with HER2-positive metastatic breast cancer conducted in accordance with the International Council for Harmonisation Guidance for Industry E6 Good Clinical Practice, the Declaration of Helsinki, and applicable local regulatory requirements. All patients provided written informed consent before starting any study-related procedures. The full trial protocol and all other relevant study documentation were approved by the institutional review board or ethics committee at each study center before study initiation.

### Eligibility

Full inclusion and exclusion criteria have been previously reported [16]. Eligible patients were adults with histologically confirmed HER2-positive breast cancer having ≥ 1 measurable metastatic target lesion. Key eligibility criteria included an Eastern Cooperative Oncology Group performance status of 0 to 2 and left ventricular ejection fraction (LVEF) within normal range [16]. Patients must not have received chemotherapy or HER2-targeted therapy within 1 year of diagnosis of metastatic disease.

### Study design

Details of the study design and dosing schedules have been previously reported (Online Resource 1) [16]. Briefly, patients were randomly assigned 1:1 to receive taxane of institutional choice (docetaxel or paclitaxel) plus trastuzumab-dkst or trastuzumab for 8 cycles (24 weeks). Patients with at least stable disease as defined by Response Evaluation Criteria in Solid Tumors version 1.1 (RECIST v1.1) at week 24 could continue with monotherapy mAb treatment according to their original randomization until progression, discontinuation due to unacceptable toxicity, or death. At the end of treatment, patients were followed every 3 months for 36 months from the date of randomization or death to assess survival. The secondary objective of this phase 3 study was to assess OS at 36 months or after 240 deaths, whichever occurred first, as observed from the time of randomization of the last patient.

### Efficacy evaluation

Overall survival was defined as the time from randomization to date of death due to any cause and was cumulative through 36 months of follow-up. The endpoints for the primary and secondary study objectives (ie, ORR, PFS) were analyzed at week 24 for the combination therapy phase and at week 48 for the monotherapy phase, and have been previously reported [16, 19]. In this analysis, efficacy endpoints were reported in all patients who enrolled after the second protocol amendment (intention-to-treat population [ITT]), which excluded patients who had already received first-line therapy.

### Safety

The safety population included all patients who received ≥ 1 dose of trastuzumab-dkst or trastuzumab. Assessment of treatment-emergent adverse events (TEAEs) included type, incidence, severity (graded by the National Cancer Institute Common Terminology Criteria for Adverse Events, version 4.03), timing, seriousness, and relatedness. Safety analyses also included assessment of LVEF values. Safety during combination and monotherapy has been previously reported [16, 19]. This report includes assessment of any updates observed through the final analysis, including accumulated data on AEs of special interest.

### Statistical analysis

Details on sample size have been previously reported [16]. Clinical activity was evaluated by assessing progression of disease, defined according to RECIST v1.1. Descriptive statistics were used to summarize patient disposition, baseline characteristics, and treatment administration, and SAS® software version 9.2 or later (SAS, Cary, NC) was used for analysis. For OS and PFS, Kaplan–Meier plots by treatment group were presented, and the log-rank test of the 2 groups unadjusted for covariates was performed.

## Results

### Patient disposition and baseline characteristics

**Fig. 1:**
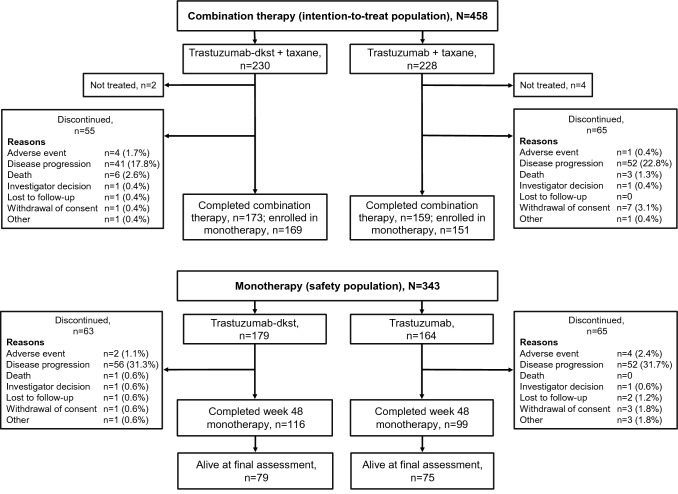
Patient CONSORT diagram for the intention-to-treat population in the HERITAGE trial

Of the 500 randomized patients, 249 were randomized to receive trastuzumab-dkst and 251 were randomized to receive trastuzumab, each in combination with taxane. The ITT population, used to evaluate efficacy, was composed of 458 female patients (230 randomized to trastuzumab-dkst and 228 randomized to trastuzumab) who had not previously received first-line therapy (Fig. 1). The mean (SD) age of patients was slightly lower in the trastuzumab group (52.9 ± 11.2) than in the trastuzumab-dkst group (54.3 ± 11.0). Demographics and baseline characteristics for both treatment groups were similar with respect to age, race, height, weight, body surface area, exposure to prior therapies, and time since diagnosis and were unchanged compared with the week 48 analyses (Table 1) [19]. The safety population was composed of 493 patients, defined as those who received ≥ 1 dose of study drug, regardless of whether they had received prior first-line therapy.

**Table 1:** Demographics and baseline characteristics: ITT population

Parameter	Patients, *n* (%)	
	Trastuzumab-dkst + taxane (*N* = 230)	Trastuzumab + taxane (*N* = 228)
Age, mean (SD), years	54.3 (11.0)	52.9 (11.2)
Race, *n* (%)		
Asian	70 (30.4)	72 (31.6)
Black or African American	1 (0.4)	2 (0.9)
White	159 (69.1)	154 (67.5)
Height, mean (SD), cm	159.0 (7.1)	159.3 (7.6)
Weight, mean (SD), kg	68.4 (15.0)	68.9 (16.0)
BSA, mean (SD), m^2^	1.73 (0.21)	1.73 (0.22)
Assigned taxane, *n* (%)		
Docetaxel	193 (83.9)	192 (84.2)
Paclitaxel	35 (15.2)	32 (14.0)
No treatment	2 (0.9)	4 (1.8)
ER + or PR + , *n* (%)	102 (44.3)	101 (44.3)
Prior treatment, *n* (%)		
Trastuzumab	22 (9.6)	16 (7.0)
Taxane	46 (20.0)	42 (18.4)
Time from diagnosis to metastatic disease, *n* (%)		
< 2 years	146 (63.5)	153 (67.1)
≥ 2 years	75 (32.6)	71 (31.1)

*BSA* body surface area, *ER* + estrogen receptor–positive, *PR* + progesterone receptor–positive

After combination therapy, 343 patients entered the monotherapy phase for safety analysis (trastuzumab-dkst, *N* = 179; trastuzumab, *N* = 164). The demographic profile for patients with safety monotherapy data is consistent with that for the ITT efficacy population. At the time of the final OS analysis, 169 patients in the safety population had received further lines of therapy (Online Resource 2), with similar distribution of HER2-targeted treatments (15.6% [*n* = 28] vs 18.9% [*n* = 31]), endocrine therapies (9.5% [*n* = 17] vs 14.6% [*n* = 24]), and chemotherapies (30.2% [*n* = 54] vs 25.0% [*n* = 41]) in the trastuzumab-dkst and trastuzumab groups, respectively.

### Efficacy evaluation

**Fig. 2:**
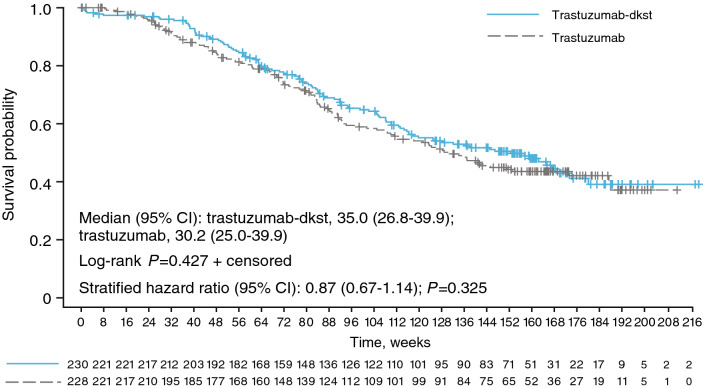
Kaplan–Meier plot of overall survival at the final assessment in the intention-to-treat population based on central tumor evaluation. Numbers of patients at risk are displayed at the bottom of the figure. The stratified hazard was calculated by assigned taxane, tumor progression, and tumor endocrine status

At the time of final analysis, 242 patients had died during the study: 116 patients treated with trastuzumab-dkst, 124 patients treated with trastuzumab, and 2 untreated patients (1 patient from each group). At final assessment, 121 (52.6%) patients were alive in the trastuzumab-dkst group, and 114 (50.0%) patients were alive in the trastuzumab group (including 79 [trastuzumab-dkst] and 75 [trastuzumab] patients who received monotherapy during the study). The survival curves did not significantly differ between treatment groups (*P* = 0.427; Fig. 2). The median OS by Kaplan–Meier analysis was 35.0 months in the trastuzumab-dkst group and 30.2 months in the trastuzumab group. Overall median follow-up time was 32.6 months (35.7 months in the trastuzumab-dkst group and 31.1 months in the trastuzumab group). The 95% CI of the hazard ratio (HR) for OS included “1” for both trastuzumab-dkst and trastuzumab at the time of the final analysis, indicating no relevant differences in survival benefit between treatment groups.

**Fig. 3:**
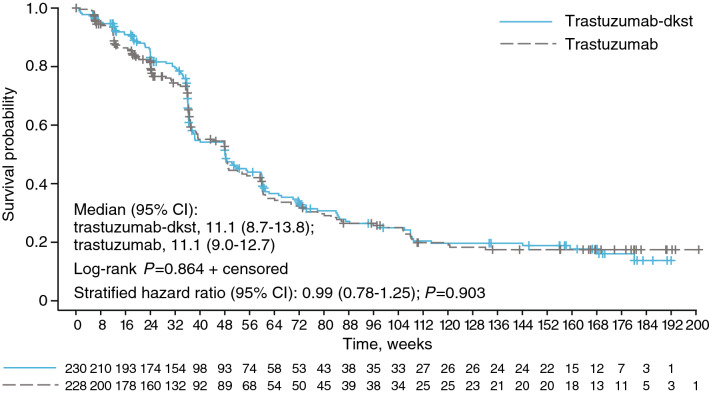
Kaplan–Meier plot of progression-free survival at the final assessment in the intention-to-treat population based on central tumor evaluation. Numbers of patients at risk are displayed at the bottom of the figure. The stratified hazard was calculated by assigned taxane, tumor progression, and tumor endocrine status

At final assessment, 82 (35.7%) patients in the trastuzumab-dkst group were free of progression compared with 86 (37.7%) patients in the trastuzumab group. The time-to-event curves for both treatment groups were not statistically significantly different (*P* = 0.864; Fig. 3). As with OS, the 95% CI of the OS ratio (trastuzumab-dkst to trastuzumab) included “1” for all subgroups at the time of the final analysis, and hence, no relevant differences between subgroups were observed. However, tumor endocrine status (negative vs positive; HR 1.40; *P* = 0.017), race (Black vs White and Asian vs White; HR 3.71 and 1.38, respectively; *P* = 0.011), previous adjuvant/neoadjuvant chemotherapy/HER2-targeted therapy (yes vs no; HR 1.39; *P* = 0.016), and number of metastatic sites (2 vs 1, 3 vs 1, and ≥ 4 vs 1; HR 1.44, 1.51, and 2.46, respectively; *P* < 0.001) had an effect on OS. The treatment HR (95% CI) adjusted for these factors was 0.85 (0.653–1.116; *P* = 0.248). Median PFS was 11.1 months in both treatment groups. Duration of response was also not statistically different between treatment groups (*P* = 0.771), with 123 (64.1%) patients in the trastuzumab-dkst group having tumor progression or dying before the final analysis, compared with 119 (64.7%) patients in the trastuzumab group. Median DR was 9.9 months in the trastuzumab-dkst group and 9.8 months in the trastuzumab group. Sensitivity analyses of all enrolled patients showed that OS, PFS, and DR results were similar to those observed in the ITT population that included patients who had received prior first-line therapy.

### Safety and tolerability

Safety and tolerability analyses for the combination therapy and monotherapy phases of the trial were previously reported [16, 19]. From the initiation of the monotherapy phase through final analysis, the number of patients with ≥ 1 TEAE was similar between the trastuzumab-dkst (69.3% [*n* = 124]) and trastuzumab groups (72.6% [*n* = 119]). Most TEAEs occurred in similar numbers in the trastuzumab-dkst and trastuzumab groups, except for patients experiencing anemia (3.4% [*n* = 6] and 10.4% [*n* = 17], respectively), and were generally grade 1 or 2 in severity (Table 2). Overall incidence of serious AEs (SAEs) reported from the start of the monotherapy phase through the end of the trial was 5.8% (*n* = 20). Incidence of SAEs was similar between the trastuzumab-dkst and trastuzumab groups (5.6% [*n* = 10] and 6.1% [*n* = 10], respectively). Rates of treatment discontinuation due to TEAEs were similar between the trastuzumab-dkst and trastuzumab groups (3.9% [*n* = 7] and 7.3% [*n* = 12], respectively). Overall, 8 (2.3%) patients withdrew from the study because of 1 or more TEAEs, 4 (2.2%) in the trastuzumab-dkst group and 4 (2.4%) in the trastuzumab group. Throughout the entire study, 57 patients (23.1%) in the trastuzumab-dkst group and 58 patients (23.6%) in the trastuzumab group reported TEAEs of special interest, including infusion reactions (6.9% [*n* = 17], 4.9% [*n* = 12]) and hypersensitivity events (2.4% [*n* = 6], 3.7% [*n* = 9]). Few patients in each treatment group had observed LVEF values of < 50% at least once post-baseline (trastuzumab-dkst, 4.5% [*n* = 11]; trastuzumab, 3.3% [*n* = 8]).

**Table 2:** TEAEs in patients who continued on monotherapy (≥ 5% in Either Treatment Group)

TEAEs, *n* (%)	Patients, *n* (%)			
	Trastuzumab-dkst (*N* = 179)		Trastuzumab (*N* = 164)	
	All grades	Grade ≥ 3	All grades	Grade ≥ 3
Any TEAE	124 (69.3)	23 (12.8)	119 (72.6)	31 (18.9)
Headache	19 (10.6)	0	23 (14.0)	4 (2.4)
Hypertension	12 (6.7)	2 (1.1)	9 (5.5)	1 (0.6)
Increased ALT	11 (6.1)	2 (1.1)	7 (4.3)	3 (1.8)
Vomiting	10 (5.6)	0	7 (4.3)	0
Decreased ejection fraction	10 (5.6)	0	6 (3.7)	1 (0.6)
Upper respiratory tract infection	10 (5.6)	0	4 (2.4)	0
Increased AST	9 (5.0)	1 (0.6)	7 (4.3)	3 (1.8)
Fatigue	9 (5.0)	0	7 (4.3)	0
Arthralgia	9 (5.0)	0	3 (1.8)	0
Cough	9 (5.0)	0	3 (1.8)	0
Anemia	6 (3.4)	0	17 (10.4)	2 (1.2)
Peripheral neuropathy	5 (2.8)	0	9 (5.5)	0

*ALT* alanine aminotransferase, *AST* aspartate aminotransferase

As previously reported, only 2 fatal TEAEs were reported in the monotherapy phase, both unrelated to the study drug [19]. There were no additional fatal TEAEs after week 48 and no new safety concerns reported through the end of the survival follow-up.

## Discussion

Previously reported efficacy analyses from the HERITAGE trial have demonstrated similarity between reference trastuzumab and trastuzumab-dkst by comparing the endpoints OS, PFS, DR, and time to progression over 48 weeks [19]. Additionally, these studies showed a strong positive correlation between ORR at week 24 and PFS at week 48. Incidence and nature of TEAEs and SAEs were also similar between the trastuzumab-dkst and trastuzumab groups through 48 weeks [19]. After these analyses, patients were followed every 3 months for 36 months to allow for assessment of OS. This is the first phase 3 trial of a trastuzumab biosimilar to report long-term survival data similar to those for originator trastuzumab in patients with metastatic breast cancer, confirming the use of a short-term endpoint (ORR) in a sensitive population to define and determine biosimilarity.

The comparable OS between the trastuzumab-dkst (35.0 months) and originator trastuzumab (30.2 months) treatment groups further supports previously reported similarity between the safety and efficacy profiles of the biosimilar and reference agents [19]. At the final assessment, approximately 50% of patients in each treatment group were alive, and survival curves for both groups were not significantly different (*P* = 0.427). These results support the long-term use of biosimilar trastuzumab in the metastatic setting. Recent evidence has also demonstrated the long-term efficacy of another trastuzumab biosimilar (SB3) in the neoadjuvant setting [20]. A phase 3 extension study aimed to assess long-term survival in patients with HER2-positive early breast cancer treated with SB3 or originator trastuzumab over 5 years. At the 3-year follow-up, the study reported similar OS rates between the biosimilar (97.0%) and originator (92.9%) trastuzumab treatment groups [20].

At the end of the study, all patients still on monotherapy were offered continued therapy and access to trastuzumab-dkst. Additional use of cancer treatments in both groups was similar. At 36 months, 169 of 343 patients in the safety population receiving monotherapy had received further lines of therapy, with similar distribution of HER2-targeted treatments between groups. However, the overall use of further lines of therapy was low, possibly due to limited accessibility and cost. It is possible that this limited use of further lines of treatment may help to explain the relatively lower OS observed in the trastuzumab-dkst and trastuzumab groups in this study compared with OS reported in recent publications [21, 22]. For example, in the CLEOPATRA trial assessing OS in patients with HER2-positive metastatic breast cancer receiving trastuzumab combined with either pertuzumab plus chemotherapy or placebo plus chemotherapy, median OS in patients receiving trastuzumab combined with placebo plus chemotherapy was 40.8 months [21]. In another previously published, prospective study of first-line therapy with trastuzumab plus chemotherapy in patients with HER2-positive metastatic breast cancer, median OS was 37.1 months [22].

In addition to comparable OS between the trastuzumab-dkst and trastuzumab groups observed in the present study, at the time of final analysis, other efficacy endpoints, including PFS and DR, demonstrated similarity. Together, these results are consistent with previous reports from the primary analysis at 24 weeks and further support the conclusion of therapeutic equivalence [16].

Safety results from the primary analysis indicated that there were no notable differences between the trastuzumab-dkst and trastuzumab treatment groups in incidence or severity of TEAEs [16, 19]. Safety profiles remained similar over the 48-week monotherapy phase and through the long-term assessment, with no new safety concerns observed [19].

Limitations of the HERITAGE trial are consistent with other biosimilar clinical development programs, including the use of a short-term primary efficacy endpoint to initially assess similarity between trastuzumab-dkst and reference trastuzumab. Assessment of ORR at 24 weeks was chosen as the primary endpoint as a short-term measure of clinical activity and safety related to the use of trastuzumab-dkst as first-line therapy for metastatic breast cancer. The long-term assessment of OS and safety builds upon previously reported efficacy results and supports the use of trastuzumab-dkst in patients with HER2-positive metastatic breast cancer. However, the summary of the secondary endpoints must be interpreted with caution as the analysis was not statistically powered. The *P* values presented for subgroup and secondary analyses should therefore be considered as a flagging indicator to show the differences among the collected data. The study was powered to determine equivalence between trastuzumab-dkst and reference trastuzumab at 24 weeks.

Biosimilars play a key role in reducing healthcare costs and improving patient access to life-saving therapies [6]. Results from a previous study have suggested that the use of trastuzumab biosimilars compared with originator trastuzumab may lead to annual cost savings in the range of 96 to 120 million euros (~ 11%) in a country like Germany [23]. The long-term data presented here further support trastuzumab-dkst as a valuable part of the growing biosimilar market that includes 4 other trastuzumab biosimilars approved by the FDA and EMA in recent years [13]. As such, trastuzumab-dkst is a safe and efficacious treatment option for patients with HER2-positive metastatic breast cancer and metastatic gastric cancer.

## Conclusion

In patients with HER2-positive metastatic breast cancer, treatment with trastuzumab-dkst and originator trastuzumab led to similar OS cumulative through 36 months of follow-up. This is the first phase 3 trastuzumab biosimilar trial to report similar long-term survival data in metastatic breast cancer. No notable differences were observed between treatment groups in other efficacy endpoints, including PFS and DR. Furthermore, there were no clinically meaningful differences in safety profiles between treatment groups and no new safety signals observed after week 48. Together, these results further support the similarity of trastuzumab-dkst with originator trastuzumab and establish this biosimilar as a safe, effective, and affordable treatment option for patients with HER2-positive metastatic breast cancer and metastatic gastric cancer.

## Supplementary Information

Below is the link to the electronic supplementary material.

Supplementary file1 (PDF 223 kb)

Supplementary file2 (PDF 101 kb)

## Data Availability

The data sets generated during and/or analyzed during the current study are available from the corresponding author on reasonable request.
