# Supplementary material for: Final overall survival analysis of the phase 3 HERITAGE study demonstrates equivalence of trastuzumab-dkst to trastuzumab in HER2-positive metastatic breast cancer
Source: Breast Cancer Res Treat. 2021 Jun 14;188(2):369–77. doi: 10.1007/s10549-021-06197-5 (PMC8260531; doi:10.1007/s10549-021-06197-5)
Supplement: Supplementary file 1 — Supplementary file1 (PDF 223 kb) [file 10549_2021_6197_MOESM1_ESM.pdf]

Article title: Final Overall Survival Analysis of the Phase 3 HERITAGE Study Demonstrates Equivalence of Trastuzumab-dkst to Trastuzumab in HER2-Positive Metastatic Breast Cancer

Journal name: *Breast Cancer Research and Treatment*

Authors: Hope S. Rugo,[1] Eduardo J. Pennella,[2] Unmesh Gopalakrishnan,[3] Miguel Hernandez-Bronchud,[4] Jay Herson,[5] Hans Friedrich Koch,[6] Subramanian Loganathan,[7] Sarika Deodhar,[7] Ashwani Marwah,[7] Alexey Manikhas,[8] Igor Bondarenko,[9] Guzel Mukhametshina,[10] Gia Nemsadze,[11] Joseph D. Parra,[12] Maria Luisa T. Abesamis-Tiambeng,[13] Kakhaber Baramidze,[14] Charuwan Akewanlop,[15] Ihor Vynnychenko,[16] Virote Sriuranpong,[17] Gopichand Mamillapalli,[18] Sirshendu Roy,[19] Eduardo Patricio Yanez Ruiz,[20] Abhijit Barve,[2] Adolfo Fuentes-Alburo,[2] Cornelius F. Waller[21]

Corresponding author: Hope S. Rugo, Professor of Medicine, University of California San Francisco Helen Diller Family Comprehensive Cancer Center, 1825 4th St, 3rd Floor BCC San Francisco, CA 94158, Hope.Rugo@ucsf.edu

ONLINE RESOURCE 1

Supplementary Figure. Study design.

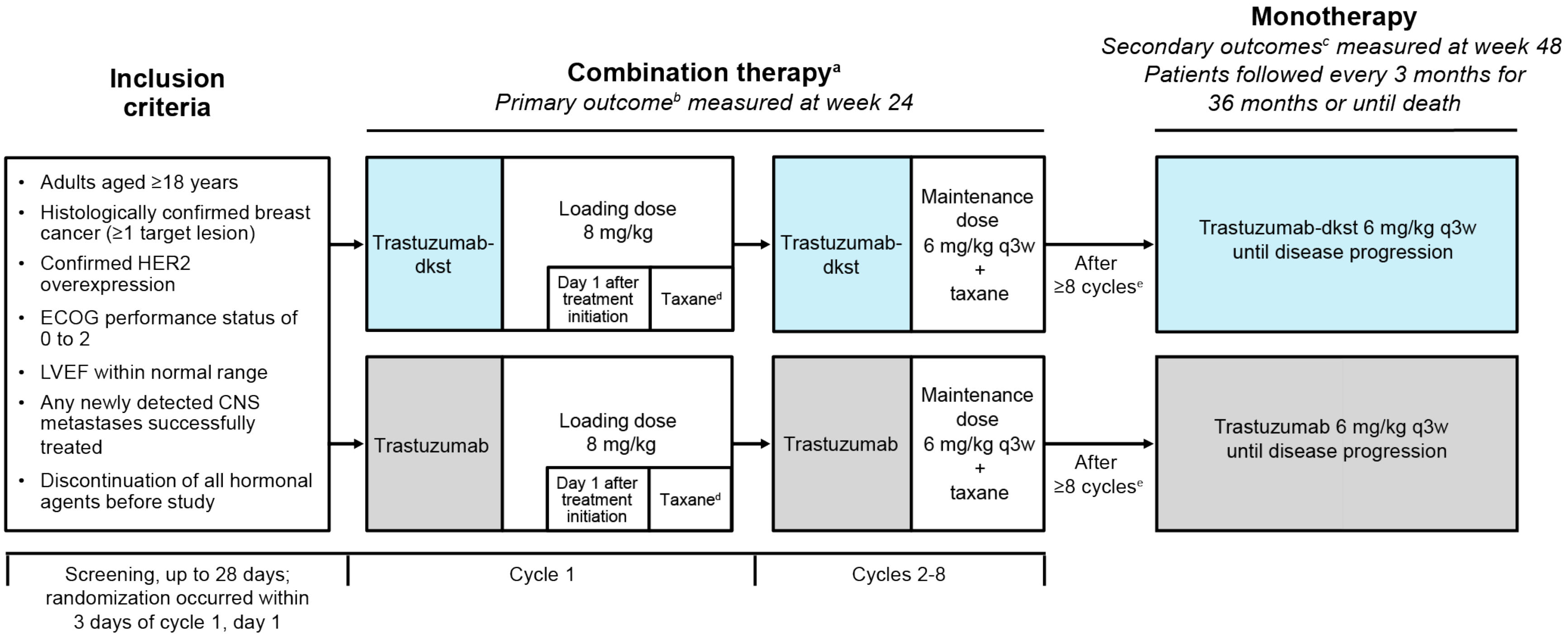

CNS, central nervous system; ECOG, Eastern Cooperative Oncology Group; LVEF, left ventricular ejection fraction; q3w, every 3 weeks. [a]Patients with stable disease could continue with combination therapy beyond 8 cycles. [b]Objective overall response rate (defined as complete or partial response). [c]Including overall survival, progression-free survival, and time to progression. [d]Taxanes consisted of either docetaxel 75 mg/m^2 q3w or paclitaxel 80 mg/m^2 weekly administered 30 minutes after treatment. [e]Patients with complete or partial response proceeded to the monotherapy

phase of the study and patients with stable disease after ≥8 cycles proceeded to the monotherapy phase of the study at the investigator's discretion. 1 cycle = 3 weeks.
