# Supplementary material for: Final overall survival analysis of the phase 3 HERITAGE study demonstrates equivalence of trastuzumab-dkst to trastuzumab in HER2-positive metastatic breast cancer
Source: Breast Cancer Res Treat. 2021 Jun 14;188(2):369–77. doi: 10.1007/s10549-021-06197-5 (PMC8260531; doi:10.1007/s10549-021-06197-5)
Supplement: Supplementary file 2 — Supplementary file2 (PDF 101 kb) [file 10549_2021_6197_MOESM2_ESM.pdf]

Article title: Final Overall Survival Analysis of the Phase 3 HERITAGE Study Demonstrates Equivalence of Trastuzumab-dkst to Trastuzumab in HER2-Positive Metastatic Breast Cancer

Journal name: *Breast Cancer Research and Treatment*

Authors: Hope S. Rugo,[1] Eduardo J. Pennella,[2] Unmesh Gopalakrishnan,[3] Miguel Hernandez-Bronchud,[4] Jay Herson,[5] Hans Friedrich Koch,[6] Subramanian Loganathan,[7] Sarika Deodhar,[7] Ashwani Marwah,[7] Alexey Manikhas,[8] Igor Bondarenko,[9] Guzel Mukhametshina,[10] Gia Nemsadze,[11] Joseph D. Parra,[12] Maria Luisa T. Abesamis-Tiambeng,[13] Kakhaber Baramidze,[14] Charuwan Akewanlop,[15] Ihor Vynnychenko,[16] Virote Sriuranpong,[17] Gopichand Mamillapalli,[18] Sirshendu Roy,[19] Eduardo Patricio Yanez Ruiz,[20] Abhijit Barve,[2] Adolfo Fuentes-Alburo,[2] Cornelius F. Waller[21]

Corresponding author: Hope S. Rugo, Professor of Medicine, University of California San Francisco Helen Diller Family Comprehensive Cancer Center, 1825 4th St, 3rd Floor BCC San Francisco, CA 94158, Hope.Rugo@ucsf.edu

ONLINE RESOURCE 2

Supplementary Table. Most Common Systemic Breast Cancer Therapies After Ending Trastuzumab

	Patients, n (%)		
**Therapy**	**Trastuzumab-dkst (N=179)**	**Trastuzumab (N=164)**	**Overall (N=343)**
At least 1 follow-up breast cancer therapy reported	90 (50.3)	79 (48.2)	169 (49.3)
Antineoplastic agents	82 (45.8)	72 (43.9)	154 (44.9)
Capecitabine	54 (30.2)	40 (24.4)	94 (27.4)
Trastuzumab	22 (12.3)	25 (15.2)	47 (13.7)
Cyclophosphamide	20 (11.2)	20 (12.2)	40 (11.7)
Lapatinib	19 (10.6)	12 (7.3)	31 (9.0)
Doxorubicin	14 (7.8)	13 (7.9)	27 (7.9)
Fluorouracil	9 (5.0)	9 (5.5)	18 (5.2)
Paclitaxel	10 (5.6)	5 (3.0)	15 (4.4)
Endocrine therapy	17 (9.5)	24 (14.6)	41 (12.0)
